# Thermal–Optical Evaluation of an Optimized Trough Solar Concentrator for an Advanced Solar-Tracking Application Using Shape Memory Alloy

**DOI:** 10.3390/ma15207110

**Published:** 2022-10-13

**Authors:** Nasir Ghazi Hariri, Kamal Mohamed Nayel, Emad Khalid Alyoubi, Ibrahim Khalil Almadani, Ibrahim Sufian Osman, Badr Ahmed Al-Qahtani

**Affiliations:** Department of Mechanical and Energy Engineering, College of Engineering, Imam Abdulrahman Bin Faisal University, P.O. Box 1982, Dammam 31441, Saudi Arabia

**Keywords:** thermomechanical, shape memory alloy, actuator, solar heat collector, thermal analysis, optical analysis, self-actuation, solar tracker, solar concentrator

## Abstract

One of the modern methods for enhancing the efficiency of photovoltaic (PV) systems is implementing a solar tracking mechanism in order to redirect PV modules toward the sun throughout the day. However, the use of solar trackers increases the system’s electrical consumption, hindering its net generated energy. In this study, a novel self-tracking solar-driven PV system is proposed. The smart solar-driven thermomechanical actuator takes advantage of a solar heat collector (SHC) device, in the form of a parabolic trough solar concentrator (PTC), and smart shape memory alloy (SMA) to produce effective mechanical energy for solar tracking applications from sun rays. Furthermore, a thermal–optical analysis is presented to evaluate the performance of the solar concentrator for the simulated weather condition of Dammam City, Saudi Arabia. The numerical results of the thermal and optical analyses show the promising feasibility of the proposed system in which SMA springs with an activation temperature between 31.09 °C and 45.15 °C can be utilized for the self-tracking operations. The work presented adds to the body of knowledge an advanced SMA-based SHC device for solar-based self-actuation systems, which enables further expansions within modern and advanced solar thermal applications.

## 1. Introduction

Continuous developments within modern and efficient technology solutions for innovative solar energy applications have attracted many researchers. Similarly, energy-related companies and manufacturers are significant players in continuously exploring alternative eco-friendly energy sources through the focus on sustainability and environmental conservation [1,2]. Environmental impact is essential for sustainable development, and its influence is enhanced by the pervasive use of fossil fuels and a lack of environmental protection [1]. Solar photovoltaic (PV) has become the principal source of electricity in several economies, where PV systems contribute significantly to total power production along with other sources, such as fossil fuels and nuclear power [3,4].

Constant investigations are being evaluated to improve the productivity and efficiency of the PV industry; correlation between systems’ operational structures and economic, social, and environmental aspects of renewable energy are investigated [5,6]. Solar cells are one type of modern technology that allows access to a clean and safe energy source since they convert solar radiation into electrical energy. Recently, the developments of effective PV tracking systems over fixed photovoltaic systems have improved the system’s overall efficiency [7].

Teng et al. [8] studied the highest power exchange of a PV tracking system, and maximum power transmission and better solar cell efficiency were achieved with a simple control circuit to match the impedance of the system’s components, enabling maximum power transmission between solar cells. Additionally, Osman, et al. [9] have studied the effect of different solar tracking systems on the performance of conventional PV modules. Their paper reveals that the dual and single trackers lead to a net energy increase compared to a fixed module—up to 29% and 19%, respectively. Moreover, the paper concluded that solar tracking systems utilize about 17% of the electrical production to run the actuators and the control system. On the other hand, Hariri et al. [10] investigated single-axis solar tracking mechanisms, in which a PV panel is moved along one axis to align it perpendicularly towards the sun’s beams throughout the day using sensor and azimuth-based methods. Experimental results showed that the sensor and azimuth-based tracking systems improved overall net energy production by about 12.68% and 7.7%, respectively. In short, most current tracking systems investigate active tracking methods that depend mainly on electrical energy, so overall energy generation remains hindered due to the tracker’s consumption.

Furthermore, shape memory alloy (SMA)-based actuators have been widely used in various areas, including robotic, automotive, aerospace, biomedical, and energy applications, because of their unique thermomechanical and shape memory effect (SME) properties. Whenever an external force deforms the SMA, it can return to its original state once heated above its activation temperature [11]. For instance, Catoor et al. [12] studied the relationship between temperature, displacement, and response speed. The result showed that the speed response and displacement change when there is an increase in the temperature. Moreover, this opens the door for more innovative and novel mechanism designs. For example, an SMA-based device has been used to enhance the aerodynamic performance of vehicles [13], generate constant force [14], power non-explosive reusable lock release mechanisms, and actuate light grippers [15,16]. Furthermore, SMAs have good chemical and physical properties and biocompatibility [17,18]. Hence, engineers and researchers across the globe show high interest in the unique properties of SMA, especially regarding developed thermomechanical energy, which it provides at a lower cost. Therefore, further innovations, designs, and systems have been proposed to integrate SMA as a smart actuation source [19,20]. Integrating the SMA within certain disciplines, such as robotic and biomedical, allows for further simplification on the design for SMA-based systems, in which it reduces the system’s volume and weight significantly, offering compact structures and systems [21].

An SMA actuator that uses temperature as a source of activation could be activated via joule heating or an external heating/cooling source, either passive or active [22]. In this study, heat transferred from the sun is the primary heating source for activating the SMA-based actuator. A solar heat collector (SHC) will be used to improve the absorb thermal energy provided by the sun. The SHC is a device used to collect the thermal energy supplied by the sun, which is stored and carried away by the flowing fluid [23]. SHCs have been used in various applications, such as domestic heating/cooling devices, storage devices, and water desalination [24,25,26]. Furthermore, some criteria when designing an SHC include the environmental criteria standards, cost-effectiveness, and technical aspects [26,27,28]. Moreover, the efficiency of the SHC device can vary depending on the design, the fluid inside the SHC, and the material of the collector and the absorber [1,29]. Some SHC devices utilize mirrors or reflecting materials to increase the heat and the solar power for a small volume so the energy can be converted into heat and then used to generate electricity. Additionally, parabolic trough collector (PTC) technology is one of the most widely used thermal power technologies among other concentrated solar power (CSP) technologies [30]. For example, the Dubai Noor project, the most considerable solar power project established globally, contains 950 MW hybrid projects, composed of 250 MW photovoltaic and 700 MW parabolic trough power technology [31]. Due to massive land use, maintenance, and installation costs, the PTC technology has vital high and intensive capital costs. Such a complicated heating system increases the potential for the system’s failure due to the massive temperature gradient on the receiver. The mirror heats the bottom side of the receiver by focusing the highly concentrated radiation intensity. In contrast, only direct solar radiation heats the top side, making the receiver’s bottom side heated more than the top side; this uneven temperature on the receiver’s outer surface causes high thermal stresses on the receiver side [30,31,32]. The temperature variance is considered the primary cause of failure, such as breakage of the receiver’s glass envelope, vacuum leakage or bending within the structure, hence causing optical losses due to the misplacement of the absorber tube from the optimal focal area. Moreover, when the receiver becomes damaged frequently and affects other components, the plant’s cost will also be affected. Therefore, with regards to the receiver’s price and reliability, it should be able to function efficiently and work reliably and economically [30,31,32,33].

In a recent research effort presented by some of the authors, a solar-driven thermomechanical actuator have been proposed [1,34,35]. The proposed actuator has shown a thermal to mechanical conversion efficiency of about 19.15%, where the actuator produces about 150 N and 130 mm [34,35]. Additionally, the authors have studied the thermal profile in the studied are of Dammam, Kingdom of Saudi Arabia (KSA) details and found that the actuator would properly operates throughout the year and does not require any maintenance routine [1].

Optimizing the parabolic trough collector is a significant step; researchers simulate and model the system to investigate the system’s effect and thermal performance before construction. There is a unique approach to overcoming the problem of interception rate and optical efficiency. The first method is to absorb more sun rays on the absorber tube using a larger diameter [36]. This is employed, for example, by Abengoa Company (Seville, Spain), where an extensive commercial concentrator with an absorber tube of 80 mm and 8.2 m aperture width is constructed. This system has been used in the Dubai Noor project, resulting in a lower price of 7.3 cents/kWh [31]. On the other hand, an alternative method is to add another reflector layer inside the glass cover on the receiver tube. The secondary reflector is used to reflect the sun rays to enhance the factor of interception when a large aperture is used with a small absorber tube [37]. Additionally, the third method is changing the absorber’s shape; hence, by adding a secondary reflector, the study made by Gong et al. [38] displayed an optical efficiency of less than 75%. Therefore, a semicircular receiver tube with a solar radiation flux distribution is used on the circular receiver tube model to intercept more sun rays. A PTC used a semicircular receiver tube with an 80° half-rim angle and 8 m aperture width with an optical efficiency of about 79.2%.

On the other hand, the integration of thermomechanical alloy, in the shape of an SMA actuator, into self-actuation and tracking mechanisms via utilizing mainly solar energy to enhance the overall performance of PV systems has not been addressed. Therefore, in recent research efforts presented by the authors, a solar-driven thermomechanical actuator has been proposed [1,34,35]. The proposed actuator has shown a thermal-to-mechanical conversion efficiency of about 19.15%, and the actuator produces about 150 N and 130 mm [34,35]. Additionally, the authors have studied the thermal profile in the studied area of Dammam, Kingdom of Saudi Arabia (KSA), where it was found that the actuator would adequately operate throughout the year and requires no maintenance routine [1]. Therefore, this study aims to add to the body of scientific knowledge an innovative methodology for a technology solution of a novel thermomechanical SMA actuator targeting solar-based self-tracking PV applications.

## 2. Materials and Methods

### 2.1. Overview

The proposed research study focuses on a thermomechanical SMA actuator’s thermal and optical performances. The thermomechanical SMA actuator has three primary elements: SMA springs, SHC, and PTC. These three elements significantly impact the actuator and, therefore, were analyzed and studied. The SHC and the PTC are placed in the actuator to increase the temperature of the SMA springs, while the activation temperature of the SMA springs is the crucial factor in the actuation mechanism. To ensure the temperature inside the SHC reaches the activation temperature of the SMA, a comprehensive study was proposed using computational fluid dynamics (CFD) simulation software version 5.6.

### 2.2. SMA Actuator Conceptual Design

**Figure 1:**
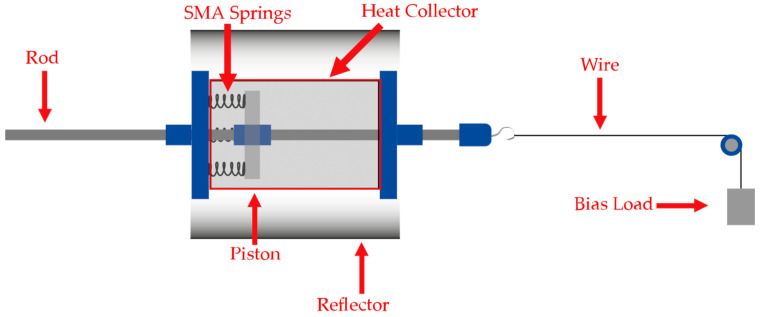
Conceptual design of the solar-driven SMA thermomechanical actuator.

The proposed research aimed to design and simulate an SMA solar-driven thermomechanical actuator. The configuration for the main parts of the proposed design is illustrated in Figure 1. The actuator is a piston-based linear actuator in which several SMA springs are arranged horizontally inside a SHC with a movable rod to ensure the linearity of the piston movement. The design utilizes the SME in the SMA springs by absorbing the heat and the intensive radiation reflected by PTC into the SHC. At sunrise, the SHC absorbs the solar radiation from the sun in addition to the reflected radiation from the PTC. The solar radiation collected through the sun and the PTC in the enclosed SHC leads to a greenhouse effect inside the SHC, raising its inner temperature, which allows the SMA springs to reach the activation temperature that triggers their SME. On the other hand, as the SMA springs lose thermal energy, the springs’ temperature drops, and the bias load stretches the SMA springs back to their original shape.

**Figure 2:**
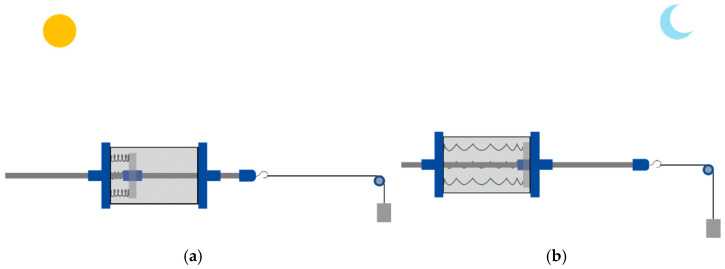
SMA-based actuator phases; (**a**) activated and (**b**) deactivated arrangements.

The solar-driven thermomechanical SMA-based actuator is a passive actuator that activates using solar thermal energy given by the sun. In addition, the actuator has two actuation phases based on the SMA states—the activation and deactivation phases—as shown in Figure 2. The activation phase occurs when the SMA springs gain thermal energy that has been absorbed via the SHC, causing the movement of the piston; this movement allows the system to lift the bias load. Conversely, when the SMA springs lose thermal energy, they deactivate, and the bias load stretches the SMA springs back to their original shape.

The designed actuator has three main limitations, which are environmental, mechanical, and thermal, and these factors can affect the feasibility of the proposed design. The environmental limitations are environment-dependent constraints, such as ambient temperature, cloudiness, and solar irradiance. Mechanical limitations include the mechanical properties of the available SMA, such as the allowable strain and the produced force. Regarding thermal limitations, the constraints of the SMA include activation temperature, deactivation temperature, and thermal endurance. All of the limitations mentioned have an effect on the performance and feasibility of the design.

### 2.3. SMA Solar Self-Tracker Conceptual Design

**Figure 3:**
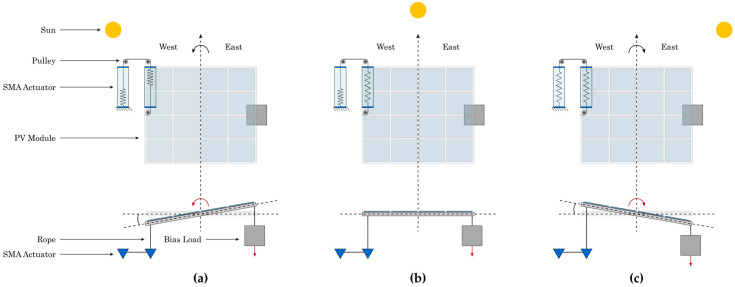
Thermomechanical actuator (**a**) before sunset, (**b**) at noon, and (**c**) after sunrise.

The proposed solution integrates two identical SMA-based, actuators allowing the system to orient the PV module into three different positions, as shown in Figure 3. During sunrise, the PV module will be oriented at an angle, casting shade on both SMA-based actuators. Both actuators will be deactivated since the internal SHC temperature is lower than the activation temperature of the SMA. During noon, one of the actuators will be exposed to the sun’s radiation, allowing the internal temperature to reach the activation temperature of the SMA. The other one will be shaded, causing the PV to be fixed horizontally. Lastly, at sunset, both SMA-based actuators will be exposed to solar radiation, activating both actuators and allowing the PV to tilt at an angle.

**Figure 4:**
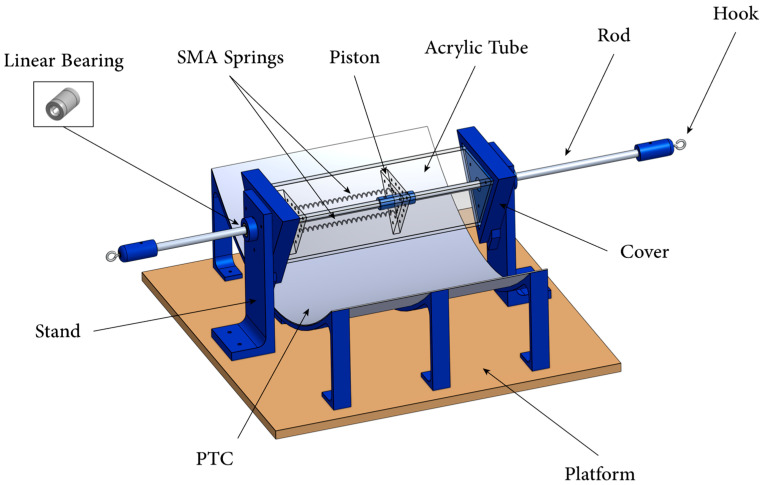
CAD model of the designed thermomechanical SMA actuator.

In order to ensure that the design of the thermal SMA actuator is aligned and functional, a three-dimensional (3D) model has been developed using computer-aided design (CAD) software. The thermal SMA actuator consists of seven main parts, including SMA springs, SHC, piston, PTC, bias load, rod, and stand holders, as highlighted in Figure 4. One end of the SMA springs is connected to the cover of the SHC, and the other end is connected to the piston to assure the linear movement of the actuator. The piston is attached to the rod, while the rod is set inside a linear ball bearing. Three linear ball bearings are attached inside the cover and the stand holders’ holes to minimize friction and guide the linear motion along a single axis. The SHC is responsible for absorbing the thermal heat from the sun, the PTC, and the surroundings, subsequently raising the temperature inside the actuator significantly above the ambient temperature. The PTC is placed to increase the thermal energy absorbed by the bottom side of the SHC by reflecting the sun’s rays.

### 2.4. Analytical Thermal Model

**Figure 5:**
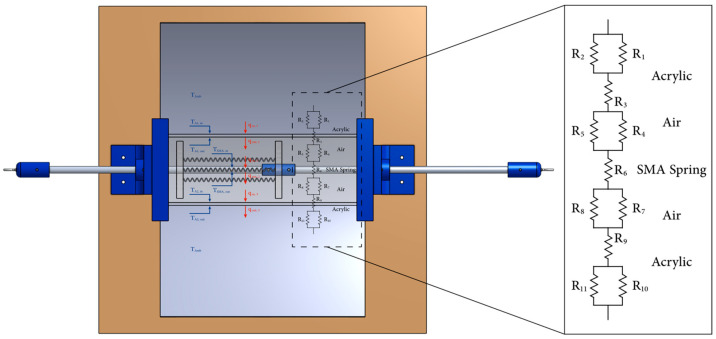
Thermal resistance for the thermomechanical SMA actuator.

The thermomechanical SMA actuator is designed to be a solar-driven actuator, and temperature is a significant key in this process. To understand the spatial variation of the temperature gradient, a simplified study for the thermal circuits is provided to highlight the heat transfer process flows under one-dimensional (1D) and steady-state conditions. As illustrated in Figure 5, the temperature inside the thermomechanical SMA actuator increases as the SHC absorbs the solar energy from the sun, the ambient weather, and the rays reflected through the PTC.

The SMA’s temperature can be obtained by analyzing the thermal circuit illustrated in Figure 5. Equation (1) is the general formula for the heat and transfer process, in which varying thermal resistance is applied depending on the heat transfer process, as shown in Equations (2)–(4) [1,34,35].

(1) $$q=\frac{\Delta \;T}{R}$$

(2) $$R_{cond}=\frac{L}{K}$$

(3) $$R_{conv}=\frac{1}{h_{conv}}\;$$

(4) $$R_{rad}=\frac{1}{h_{rad}}$$

where q represents the amount of heat transferred per unit length [W/m^2^], ΔT is the temperature difference in [K], while R represents the thermal heat transfer resistance in [K/W], and the thermal resistance for conductive, convective, and radiative varies for $R_{cond}$, $R_{conv}$, and $R_{rad}$ in [K/W]. L is the length in [m], K is the thermal conductivity in [W/m × K], $h_{conv}$ is the convective heat transfer coefficient in [W/m^2^ × K], and $h_{rad}$ is the radiative heat transfer coefficient in [W/m^2^ × K].

The acrylic plate of the SHC absorbs the thermal heat coming from the direct sun rays, the reflected rays through the PTC, and the ambient weather in the form of radiation and as a convection heat transfer process, as described in Equation (5). Furthermore, the acrylic plate will release the heat as conduction, convection, and radiation, as shown in Equation (6). In addition, the SMA springs will gain heat through radiation and convection while losing some heat throughout conduction, convection, and radiation, as shown in Equations (7) and (8). To complete the cycle, the other acrylic plate will gain the extracted heat coming from the SMA, as shown in Equation (9). At the same time, the plate will lose thermal heat in the form of conduction, convection, and radiation, as demonstrated in Equation (10).

(5) $$q_{in,1\;}=\frac{T_{amb}-T_{A1_{in}}}{\frac{1}{h_{conv_{in}}}+\frac{1}{h_{rad_{in,1}}}}\;$$

(6) $$q_{out,1}=\frac{T_{A1_{in}}-T_{A1_{out}}}{\frac{L_{1}}{K_{1}}}+\frac{T_{A1_{out}}-T_{air,1}}{\frac{1}{h_{conv_{out,1}}}+\frac{1}{h_{rad_{out,1}}}}$$

(7) $$q_{in,2}=\frac{T_{air,1}-T_{SMA_{in}}}{\frac{1}{h_{conv_{in,2}}}+\frac{1}{h_{rad_{in,2}}\;}}$$

(8) $$q_{out,2}=\frac{T_{SMA_{in}}-T_{SMA_{out}}}{\frac{L_{2}}{K_{2}}}+\frac{T_{SMA_{out}}-T_{air,2}}{\frac{1}{h_{conv_{out,2}}}+\frac{1}{h_{rad_{out,2}}}}$$

(9) $$q_{in,3}=\frac{T_{air,2}-T_{A2_{in}}}{\frac{1}{h_{conv_{in,3}}}+\frac{1}{h_{rad_{in,3}}}}$$

(10) $$q_{out,3\;}=\frac{T_{A2_{in}}-T_{A2_{out}}}{\frac{L_{3}}{K_{3}}}+\frac{T_{A2_{out}}-T_{amb}}{\frac{1}{h_{conv_{out,3}}}+\frac{1}{h_{rad_{out,3}}}}$$

where T is the temperature and h is the heat transfer coefficient. The subscript represents the medium and the heat transfer process based on Figure 5. The aim is to obtain the SMA temperature, which could be simplified—since the SMA springs are noticeably thin, it can be assumed that the inlet temperature of the SMA springs ($T_{SMA_{in}}$) is equal to the outer temperature of the SMA springs ($T_{SMA_{out}}$).

The outlet and the inlet heat transfer for the SMA springs have been equalized, forming Equation (11). Furthermore, the thermal process is shortened in the form of resistances, as shown throughout Equations (12)–(14), which simplify Equation (11) into (15). In order to obtain the SMA temperature, Equation (15) must be solved in terms of the $T_{SMA}$, as shown in Equation (16).

(11) $$\frac{T_{air,1}-T_{SMA_{in}}}{\frac{1}{h_{conv_{in,2}}}+\frac{1}{h_{rad_{in,2}}\;}}=\frac{T_{SMA_{in}}-T_{SMA_{out}}}{\frac{L_{2}}{K_{2}}}+\frac{T_{SMA_{out}}-T_{air,2}}{\frac{1}{h_{conv_{out,2}}}+\frac{1}{h_{rad_{out,2}}}}$$

(12) $$R_{1}=\frac{1}{h_{conv_{in,2}}}+\frac{1}{h_{rad_{in,2}}}$$

(13) $$R_{2}=\frac{L_{2}}{K_{2}}$$

(14) $$R_{3}=\frac{1}{h_{conv_{out,2}}}+\frac{1}{h_{rad_{out,2}}}\;$$

(15) $$\frac{T_{air_{1}}-T_{SMA_{in}}}{R_{1}}=\frac{T_{SMA_{in}}-T_{SMA_{out}}\;}{R_{2}}+\frac{T_{SMA_{out}}-T_{air,2}}{R_{3}}$$

(16) $$T_{SMA}=\frac{T_{air,1}\times R_{3}+T_{air,2}\times R_{1}}{R_{1}+R_{3}}$$

Equation (16) is the simple form, and to expand it, the resistances (*R*_1_, *R*_2_, and *R*_3_) could be resubstituted, while the $T_{air,1}$ and $T_{air,2}$ could be evaluated in terms of the acrylic and the ambient temperature.

### 2.5. D Thermal Numerical Study Setup

**Figure 6:**
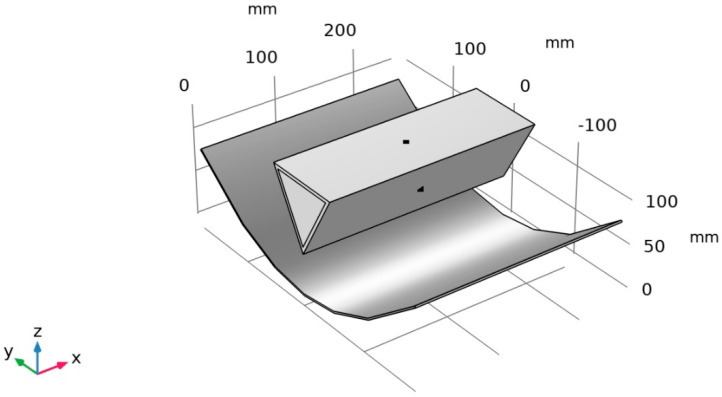
Imported CAD model of thermomechanical SMA actuator within the CFD software.

The offered thermomechanical SMA actuator contains complex thermal processes involving numerous inputs influencing the system’s thermal behavior. Therefore, a simulation-based thermal study has been conducted to understand and optimize the thermal behavior inside the SHC, aiming to reach the activation temperature of the SMA springs inside the SHC. To start the numerical study, the CAD model of the main parts of the thermomechanical SMA actuator was imported into the CFD simulation software, as shown in Figure 6. Moreover, the simulation-based study was specified to the CFD software to be performed using the actual weather conditions of the city of Dammam (26.433° N 49.8° E), KSA.

Two physical interfaces have been chosen to run the study, including the heat and transfer in solids and the surface-to-surface radiation physics interfaces. The study was performed in a time-dependent mode since the aim is to accurately understand the thermal behavior inside the SHC with respect to the studied area. Additionally, the material is assigned to be acrylic plastic for the side of the SHC, while the fluid inside is air. The thermodynamic properties of the actuator material, such as density, heat capacity at constant pressure, and thermal conductivity, are defined in Table 1. The ambient weather conditions are defined as being the same as the weather conditions of Dammam City, KSA using the meteorological data from ASHRAE 2017 that is available in the CFD software.

**Figure 7:**
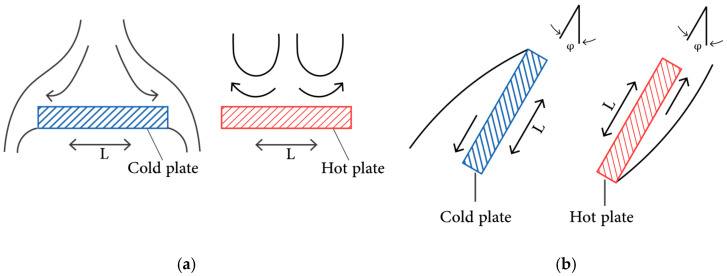
The external natural convection process for (**a**) upper horizontal plate and (**b**) inclined plate.

To integrate the CAD model with multiphysics interfacing solution tools, a set of thermal physics must be optimized, such as the heat transfer in solids and surface-to-surface radiation. The heat transfer in solids studies the heat flux due to the convection heat transfer process on the outside surface of the SHC. The convection heat process considers the change in temperature outside the SHC surface due to the ambient temperature while considering the plate surface of the SHC. The initial value conditions for the multiphysics solver are defined as the ambient temperature given in the meteorological data in Dammam City. The fluid inside the thermomechanical SMA actuator is defined as air, and all the properties are functions of the initial value condition and the ideal gas properties. The actuator has a triangular shape, and different optimization is needed for the oriented plates. The horizontal upper plate is exposed to external natural convection, as shown in Figure 7a. The other two sides of the SHC are also exposed to external natural conviction but with an inclined surface, as shown in Figure 7b.

**Figure 8:**
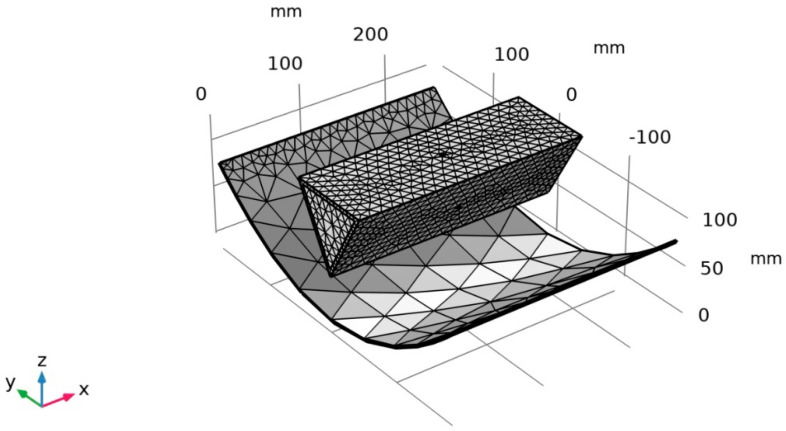
Physics-controlled mesh sequence in the multiphysics interface solution tool for the thermomechanical SMA actuator.

In addition, the PTC and the SHC have been mainly considered in the surface-to-surface radiation tools. The source of the radiative heat transfer process is mainly the sun, while the SHC side is defined as a blackbody. The initial values for the weather conditions for the Surface-to-surface radiation are set to be the ambient weather conditions. The external radiation source is the sun, with solar irradiance defined as the clear sky at noon. To resemble the mirror effect to the PTC, a diffusion mirror which will work as a mirror absorbing all the radiative coming and then radiating back in all directions has been defined. Both heat transfer in solids and surface-to-surface will be coupled in the multiphysics interface tools with a time-dependent study to investigate the thermal behavior of the actuator with the effect of the PTC. To obtain an accurate result, a physics-controlled fine mesh sequence has been applied to the thermomechanical SMA actuator, as shown in Figure 8.

## 3. Results

The results in this section highlight the thermal analysis of the SHC, including the temperature gradient, midpoint temperature, and temperature profile for different shapes. In addition, the thermal performance of the SHC has been improved based on multiple comparisons of several parameters, such as reflector presence, the shape of the SHC, dimensional optimization, and orientation. The comparisons carried out help to enhance the overall thermal performance of the SHC to ensure its compatibility with the intended application. It should be noted that all comparison simulations were run in the same period (1 July 2022).

### 3.1. Effect of Introducing a PTC on the Thermomechanical Actuator’s Thermal Behavior

**Figure 9:**
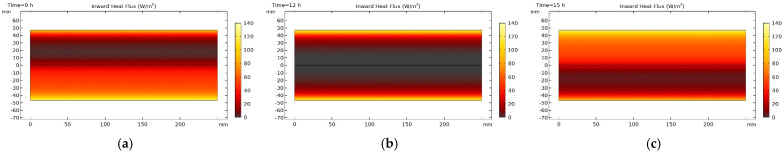
Surface plot of the inward heat flux through the projected area of the lower surface of the triangular SHC without a reflector at (**a**) 9:00; (**b**) 12:00; and (**c**) 15:00 on 1 July 2022.

In this division, the outcomes of adding a PTC to the system on the performance of the SHC are explored. Multiple comparisons are carried out to explore the feasibility of using a reflector, including inward heat flux variation on the lower surface of the SHC in different conditions. The amount of inward heat flux through the lower surface of the triangular SHC is shown in Figure 9. In the morning, when the sun rises from the east, the eastern side of the SHC surface will gain high heat flux, while the western side will be shaded, causing significantly lower inward heat flux, as illustrated in Figure 9a. At noon, the sun is perpendicular to the SHC; the upper surface casts shade, preventing the heat flux from reaching the bottom side of the SHC, as shown in Figure 9b. In Figure 9c, we can note an increase in the inward heat flux on the western side while shadows are projected on the eastern side, causing a lower inward heat flux.

**Figure 10:**
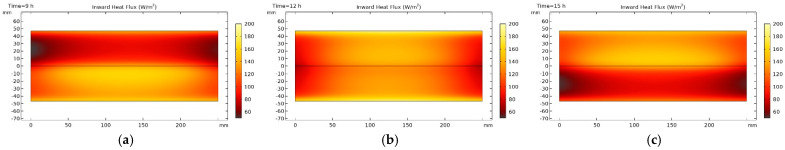
Surface plot of the inward heat flux through the projected area of the lower surface of the triangular SHC with a reflector at (**a**) 9:00; (**b**) 12:00; and (**c**) 15:00 on 1 July 2022.

To navigate the enhancement by adding the PTC, the same simulation has been conducted to study the inward heat flux on the lower surface of the SHC when adding the PTC. Figure 10 is a 2D surface plot illustrating the inward heat flux distribution on the projected lower surface area of the triangular SHC during the day. The surface plot facilitates investigation into how adding a reflector affects the performance of the SHC by redirecting the sun rays to the lower surface of the SHC during the day. For example, in the morning, the eastern side projects shadow onto the western side of the SHC, but the PTC compensates for this by reflecting the sun radiation, allowing the lower surface to receive higher heat flux and increase the coverage area, as shown in Figure 10a. By comparing Figure 9b and Figure 10b, we can see the significant improvement the PTC provides.

**Figure 11:**
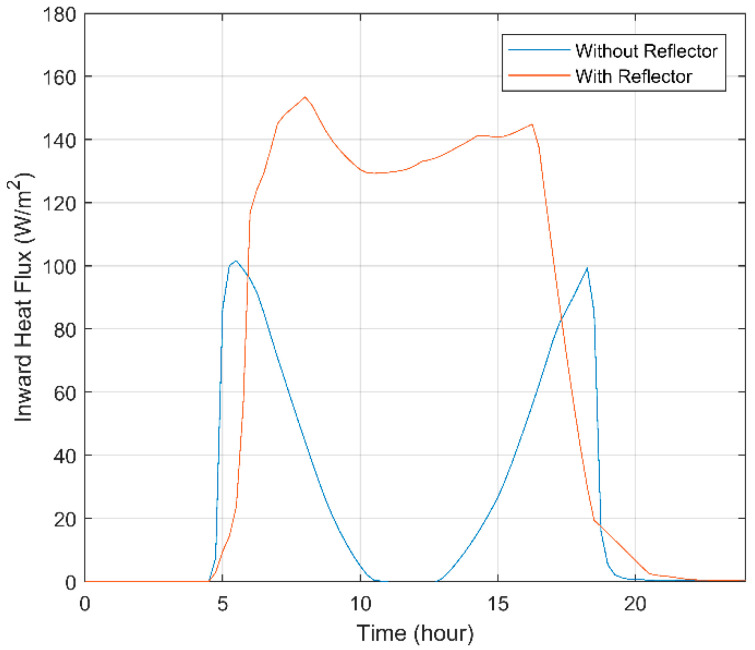
Variation in the amount of inward heat flux through the lower surface of the triangular SHC during a whole day with the effect of using the reflector.

Figure 11 demonstrates the variation in the amount of inward heat flux through the lower surface of the triangular SHC throughout the day and the effect of using a PTC. We can note that the contribution of the PTC not only increases the heat flux coverage but also highly increases the intensive heat flux on the lower surface throughout the day, as illustrated in Figure 11. In the morning, the body of the PTC casts shadows, reducing the solar radiation on the lower surface of the SHC for a period. Furthermore, at noon, the SHC without the PTC does not received any heat flux; this is incomparable to the SHC with the PTC, which almost reaches 130 [W/m^2^]. It is evident in the plot that using a reflector increases the inward heat flux, which means increasing the temperature inside the SHC, which enables the SMA springs to reach this specified activation temperature. It can be concluded by the analysis of the revealed data that the addition of a reflector increases SHC compatibility with the application aimed.

### 3.2. Thermal Behavior of a Triangular SHC in Comparison to a Circular SHC

This section evaluates the thermal behavior of different SHC to compare circular and triangular shapes. This evaluation enables better judgment of which shape to optimize the SHC functionality and feasibility for the application of a passive solar tracking system.

**Figure 12:**
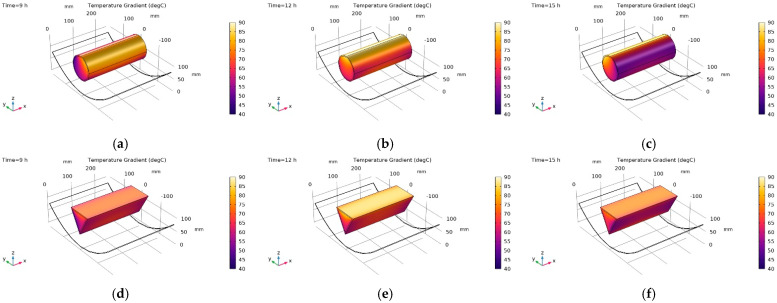
Three-dimensional plot of the temperature gradient of the circular SHC with a reflector at (**a**) 9:00, (**b**) 12:00, and (**c**) 15:00 and the temperature gradient of the triangular SHC with a reflector at (**d**) 9:00, (**e**) 12:00, and (**f**) 15:00.

One of the parameters from which the shaping capability of the SHC can be determined is the temperature gradient across the SHC. The temperature gradient across the SHC works as an indicator that the temperature is distributed evenly, allowing the temperature to reach the activation temperature along the SMA springs. Figure 12 shows a 3D plot of the different shapes’ temperature gradients. The outcome of the figure showed little to no difference between the different shapes in both temperature gradient and thermal behavior.

**Figure 13:**
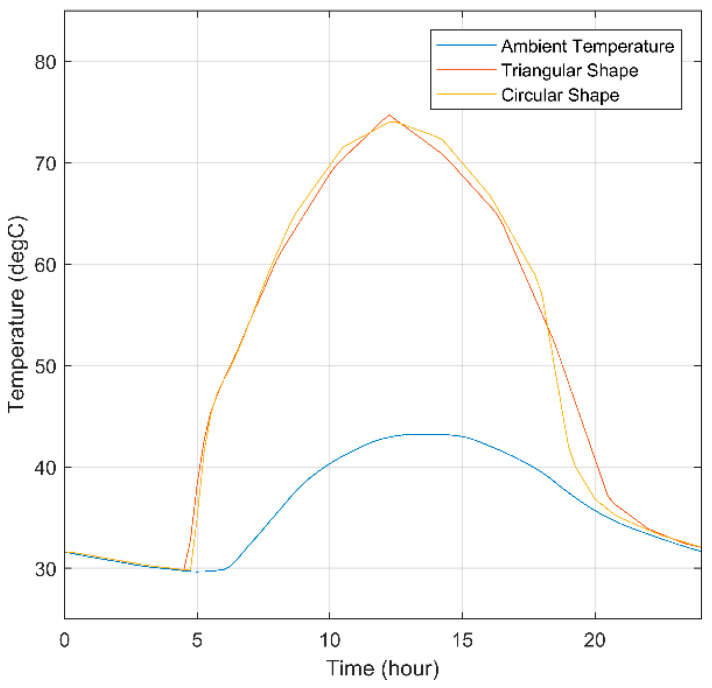
Midpoint temperature for different shapes of SHC.

Another parameter that must be evaluated is the ability to increase the temperature inside the SHC. Moreover, the temperature inside must be maintained above the SMA activation temperature during the daytime. Therefore, the distributed SMA springs (shown previously in Figure 4) have been integrated into a single point (midpoint). For simplicity, the midpoint is assumed to be the SMA temperature. In Figure 13 the midpoint temperature of both the circular and triangular-shaped SHCs are plotted. Both shapes increase the inside temperature 30 °C above the ambient temperature, reaching 74 °C. Furthermore, the two shapes maintained a midpoint temperature above the SMA activation temperature for longer. Moreover, the plot showed a similar outcome to Figure 12, in which minimum to no difference was indicated, except in Figure 13, where the outcome was dependent purely on the temperature of a single point (midpoint). The maximum, minimum, and average temperatures of the circular SHC were 74.01 °C, 29.77 °C, and 50.58 °C, respectively. Moreover, the maximum, minimum, and average temperatures of the triangular SHC were 74.69 °C, 29.84 °C, and 50.69 °C, respectively. The minimum differences in the temperatures were slightly inclined toward the triangular-shaped SHC; therefore, the triangular SHC is preferred due to the simplicity of manufacturing.

### 3.3. Optimization of the Geometrical Parameters of the Reflector

To ensure the optimum performance of the utilized reflector for increasing the temperature of the solar-driven thermomechanical SMA actuator, two main geometrical parameters are considered. The parameters conserved to optimize the designed reflector are the aperture width and the focal length. These parameters affect the performance of the PTC, which affects the system’s performance as a whole. The first parameter is the aperture width of the reflector, where a variety of aperture widths were simulated to discover how the aperture width affects the midpoint temperature of the SHC. The second parameter was the focal length between the reflector and the SHC, where multiple focal lengths were simulated.

**Figure 14:**
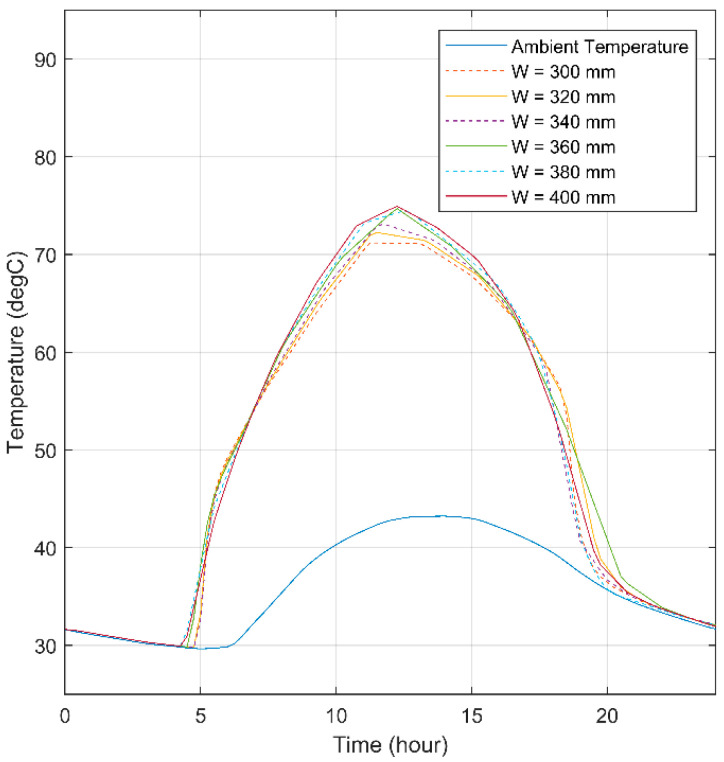
Midpoint temperature for triangular SHC for different values of aperture width.

The simulation was conducted by fixing the focal length and varying the aperture widths ranging from 300 mm to 400 mm to ensure the optimum from which the SHC reaches the highest temperature. The temperature variation of the SHC depending on aperture widths is demonstrated in Figure 14. Although the different aperture widths have the advantage of increasing the temperature depending on the time of the day, the temperature difference is minor, concluding that the aperture width can be neglected in the range mentioned.

**Figure 15:**
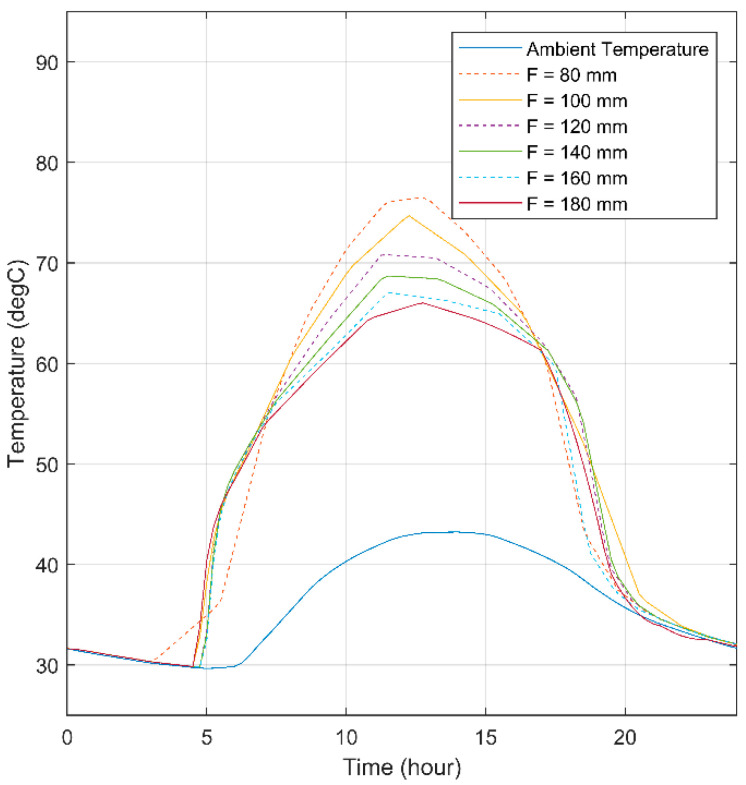
Midpoint temperature for triangular SHC for different values of focal length.

In order to optimize the focal length, the aperture width was initially fixed, while multiple focal lengths were evaluated to ensure the optimized design of the system. The midpoint temperature inside the SHC is plotted in Figure 15 under different focal lengths ranging from 80 mm to 180 mm. The results of the plotted data showed that the focal length significantly affects the midpoint temperature inside the SHC. For example, the maximum midpoint temperature at noon was 76.25 °C at a focal length of 80 mm, while the minimum midpoint temperature at noon was 65.44 °C at a focal length of 180 mm. Although the maximum midpoint temperature at noon was at a focal length of 80 mm, it was noted that a smoother temperature curve with the second highest temperature at noon was at a focal length of 100 mm with a temperature of 74.17 °C, which makes it the optimal option.

### 3.4. SHC Orientation’s Effect on the Thermal Behavior

**Figure 16:**
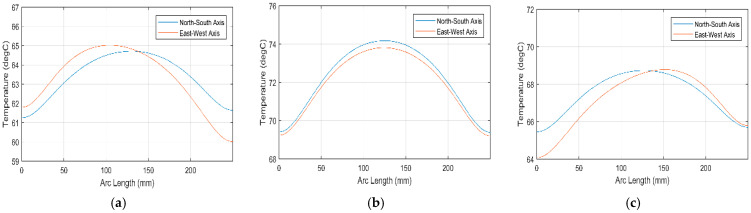
Temperature profile of the triangular SHC with a reflector at (**a**) 9:00; (**b**) 12:00; (**c**) and 15:00 on 1 July 2022.

The orientation of the SHC is essential to its temperature profile, which is why a simulation of the temperature profile under different orientations is carried out. In Figure 16 the temperature profile at the center line of the SHC is plotted for both the north–south axis and the east–west axis. It can be noted that the temperature profile inside the SHC varies depending on the orientation at 09:00 and 15:00; however, the temperature profile is similar at noon. The difference in the temperature profile depending on the orientation is due to the sun’s direction at different times of the day. Although the east–west-oriented SHC has a higher maximum temperature in Figure 16a,c, the maximum temperature at noon is higher in the north–south-oriented SHC as seen in Figure 16b. In addition, the temperature profile is more evenly distributed for the north–south orientation. Therefore, it could be concluded from the outcome of the simulation that the north–south is more fitted for the application intended since it provides a well-distributed temperature profile inside the SHC throughout the day.

### 3.5. One-Year Midpoint Temperature Variation in the Optimized SHC

**Figure 17:**
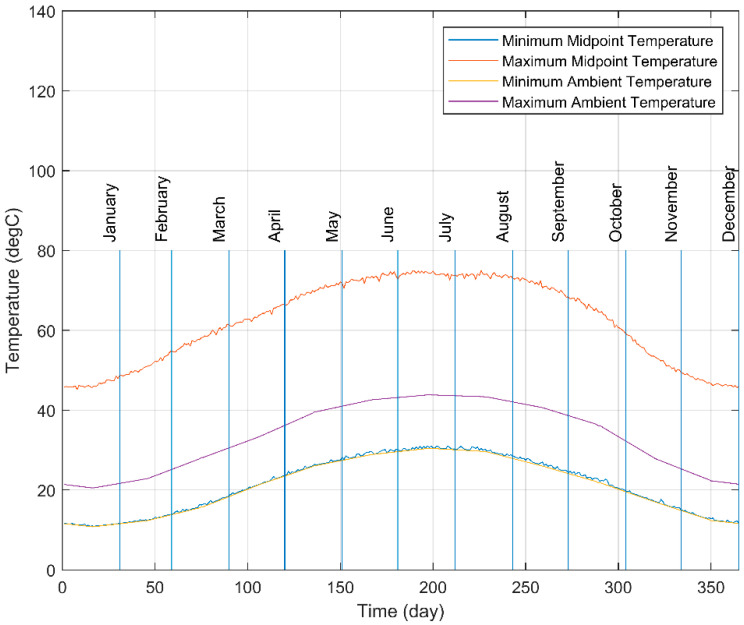
Maximum and minimum daily midpoint temperature and ambient temperature variation throughout 2022G.

In order to ensure the capability of the designed system through a single year, a simulation to compute the midpoint temperature inside the SHC was carried out. The computed midpoint temperature results of the simulation performed for a year are mapped in Figure 17. According to the simulation, the highest and lowest points of the maximum midpoint temperature line were 75.00 °C and 45.15 °C, respectively. In contrast, the highest and lowest points of the minimum midpoint temperature line were 31.09 °C and 10.89 °C, respectively. Moreover, the average midpoint temperature inside the SHC throughout the year was 39.46 °C. Therefore, it was concluded that any SMA springs with an activation temperature varying between 31.09 °C and 45.15 °C—in addition to being able to withstand a temperature of 75.00 °C without becoming plastically deformed or damaged—can be used in the designed SHC under Dammam City’s weather conditions. Additionally, the yearly temperature profile has shown a close similarity to previous efforts by Hariri, et al. [34]; however, the temperature profile of the proposed system is higher due to the utilization of such reflectors.

## 4. Conclusions

The presented article is a thermal–optical evaluation of an optimized trough solar concentrator for advanced solar-tracking applications using shape memory alloy. The main element determining the feasibility of the solar concentrator is the temperature of the SHC, from which the thermal energy would be transferred into the SMA, which would convert the heat to mechanical energy. Multiple numerical simulations were carried out to optimize the design of the SHC. The outcomes of the tests performed proved the capability of the optimized design of the SHC, and the outcomes of the simulations were summarized as follows:

- The performance of the SHC is best with the presence of a reflector with an aperture width between 300 and 400 mm and a focal length of 100 mm.
- The SHC is better oriented on the north–south axis since this orientation provides a well-distributed temperature profile inside the SHC.
- SMA springs with an activation temperature varying between 31.09 °C and 45.15 °C, in addition to being able to withstand a temperature of 75.00 °C without becoming plastically deformed or damaged, are applicable.
- The outcomes from the numerical simulation prove the feasibility of the design for the intended application.

The presented outcomes show the feasibility of innovative technology of an actuation method that drives a passive advanced solar tracker. The proposed solar tracking system is solar-driven via the utilization of an SMA-based actuator, and it is the first of its kind. Future work on the proposed system may include thermal evaluation of the system under weather conditions in different areas to test the system’s feasibility accordingly. In addition, future work may include further experimental analysis of the system and comparisons between the experimental and numerical data. Additional future work may include the shadow analysis of the PV module to ensure the optimal arrangement of the actuation mechanism.

## Figures and Tables

**Table 1:** Properties of acrylic plastic used for the SHC.

Property	Value	Unit
Heat capacity at constant pressure	1470	J/(Kg·K)
Density	1190	$\mathrm{Kg}/m^{3}$
Thermal conductivity	0.18	W/(m·K)

## Abbreviations

PV	Photovoltaic
SMA	Shape memory alloy
SME	Shape memory effect
SHC	Solar heat collector
PTC	Parabolic trough collector
CSP	Concentrated solar power
KSA	Kingdom of Saudi Arabia
3D	Three-dimensional
1D	One-dimensional
CFD	Computational fluid dynamics
**Symbols**	
q	Amount of heat transferred [W/m^2^]
T	Temperature [°C]
R	Thermal resistance [°C/W]
L	Length [m]
K	Thermal conductivity [W/m × K]
h	Heat transfer coefficient [W/m^2^ × K]
**Subscript**	
cond	Conduction heat transfer process
conv	Convection heat transfer process
rad	Radiation heat transfer process
in	Input
out	Output
A	Acrylic
amb	Ambient
